# A systematic review and network meta-analysis protocol of adjuvant chemotherapy regimens for resected gastric cancer

**DOI:** 10.1097/MD.0000000000014478

**Published:** 2019-02-15

**Authors:** Long Ge, Liangying Hou, Qingxia Yang, Yiting Wu, Xiue Shi, Jiang Li, Kehu Yang

**Affiliations:** a The First Clinical Medical College; b Evidence Based Social Science Research Center, School of Public Health; c The Second Clinical Medical College, Lanzhou University; d Institute for Evidence Based Rehabilitation Medicine of Gansu Province, Lanzhou; e National Cancer Center/National Clinical Research Center for Cancer/Cancer Hospital, Chinese Academy of Medical Sciences and Peking Union Medical College, Beijing; f Evidence-based Medicine Center, School of Basic Medical Sciences, Lanzhou University, Lanzhou, China.

**Keywords:** adjuvant chemotherapy, bayesian, gastric cancer, network meta-analysis, protocol

## Abstract

**Background:**

Gastric cancer is the third leading cause of cancer death in the world. The benefit of adjuvant chemotherapy has been demonstrated by published individual patient data meta-analysis and Cochrane systematic review. However, there is no consensus on which is the optimal adjuvant chemotherapy regimens. Present network meta-analysis aims to compare the differences of effect between all available adjuvant chemotherapy regimens in improving overall survival and disease-free survival, and to rate the certainty of evidence from present network meta-analysis.

**Methods:**

We will conduct this systematic review and network meta-analysis using Bayesian method and according to Preferred Reporting Items for Systematic review and Meta-Analysis Protocols (PRISMA-P) statement. We will search PubMed, EMBASE.com, the Cochrane Central Register of Controlled Trials (CENTRAL), Chinese National Knowledge Infrastructure (CNKI), and Chinese Biological Medical Database (CBM), and ClinicalTrials.gov (http://clinicaltrials.gov/) to identify randomized controlled trials (RCTs) comparing adjuvant chemotherapy to surgery alone. We will assess the risk of bias of individual RCTs using a modified version of Cochrane tool. We will also use the advance of GRADE to rate the certainty of network meta-analysis. Data analysis will be performed with R-3.4.1 and WinBUGS software.

**Results:**

The results of this study will be published in a peer-reviewed journal.

**Discussion:**

To the best of our knowledge, this systematic review and network meta-analysis will firstly use both direct and indirect evidence to compare the differences of all available adjuvant chemotherapy regimens for resected gastric cancer patients. This is a protocol of systematic review and meta-analysis, so the ethical approval and patient consent are not required.

## 1 Introduction

Gastric cancer is the third leading cause of cancer death in the world.^[1]^ The International Agency for Research on Cancer estimates that there will be 1.03 million newly diagnosed gastric cancer in the world in 2018 and that 0.78 million will die from the disease, of them, 40% of newly diagnosed patients and 50% of death are from China.^[1]^ Although surgical resection remains the cornerstone of treatment and the only curative treatment for patients with gastric cancer,^[2]^ 40% to 80% of patients die of disease relapse.^[3]^ Meta-analyses have shown improved survival in gastric cancer patients treated with adjuvant chemotherapy compared to those who underwent surgery alone, especially in Asia.^[4–7]^ An individual patient data meta-analysis including 17 randomized controlled trials (RCTs) demonstrated that adjuvant chemotherapy was associated with a statistically significant benefit in terms of overall survival (OS) (hazard ratio [HR], 0.82; 95% confidence interval [CI], 0.76–0.90) and disease-free survival (DFS) (HR, 0.82; 95% CI, 0.75–0.90).^[6]^ A Cochrane systematic review published in 2013 also demonstrated the benefit of adjuvant chemotherapy in resected gastric cancer.^[7]^ However, there is no consensus on which is the optimal adjuvant chemotherapy regimens. The national and international guidelines also showed inconsistent recommendations of adjutant chemotherapy. For example, ESMO clinical guidelines recommended 5-FU-based chemotherapy for patients with ≥Stage IB gastric cancer who have undergone surgery without administration of preoperative chemotherapy, and S-1 following D2 resection for Asian patients.^[8]^ While National Comprehensive Cancer Network (NCCN) gastric cancer recommended capecitabine combined with oxaliplatin following primary D2 lymph node dissection.^[9]^ Well-conducted conventional meta-analyses of RCTs are accepted as the best-quality evidence to inform clinical practice and health policy.^[10,11]^ However, it has been impractical to test all available intervention directly in a conventional meta-analysis or RCT. Network meta-analysis has become increasingly popular to evaluate healthcare interventions since it allows for estimation of the relative effectiveness among all interventions and rank ordering of the interventions even if head-to-head comparisons are lacking.^[12,13]^

Two recent network meta-analyses have focused on the relevant effective of adjuvant chemotherapy, adjuvant radiotherapy and adjuvant radiochemotherapy in resected gastric cancer, however, they failed to compare different adjuvant chemotherapy regimens.^[14,15]^ Although a network meta-analysis published in 2014 compared several adjuvant chemotherapy regimens, they failed to include some main adjuvant chemotherapy regimens such as XELOX (capecitabine + oxaliplatin) and S-1 regimens.^[16]^ In addition, there are some new trials comparing other adjuvant chemotherapy and long-term results of previous RCTs have been updated.^[17,18]^ What's more, all the results of the above network meta-analyses were summarized as relative treatment effects. However, absolute effects are more straightforward for shared clinical decision making.^[19]^ Although network meta-analysis is highly attractive, the treatment rankings and effect estimates often are uncertain and imprecise.^[20,21]^ GRADE approach should be used to rate the confidence or certainty of evidence from network meta-analysis, which could reflect the extent to which confidence in an estimate of the effect.^[22]^

Present study will systematically collect all available RCTs that compared different adjuvant chemotherapy regimens with surgery resection alone, to comprehensively compare the differences between all available adjuvant chemotherapy regimens in improving OS and DFS, and to rate the certainty of evidence from present network meta-analysis.

## 2 Methods

This protocol will be reported according to preferred reporting items for systematic review and meta-analysis protocols (PRISMA-P),^[23]^ and this network meta-analysis will be performed and reported in accordance with PRISMA Extension version (PRISMA-NMA).^[24]^ As a part of our project, this study protocol has been registered on the international prospective register of systematic review (PROSPERO) (CRD42018104782).

### 2.1 Eligibility criteria

Studies will be included in this systematic review and network meta-analysis if meet the following eligibility criteria: 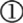 Type of participants: eligible participants are age of 18 years or older, have undergone partial or total gastrectomy, irrespective of the location of the cancer. Trials including patients with oesophagogastric junction will be excluded. 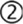 Type of design: Only RCTs focusing on different adjuvant chemotherapy for patients with resected gastric cancer. 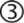 Type of interventions: surgery alone and all adjuvant chemotherapy regimens. 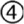 Type of outcomes: the outcomes of interest will include OS, which is defined as the time from randomization until the date of death, and DFS, which is the time from date of random assignment to date of recurrence or death. 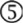 Other criteria: there are no limitations on language of publication, year of publication, publication status.

We excluded RCTs assessing the following interventions: preoperative and perioperative treatments, postoperative radiotherapy and radiochemotherapy, and immunotherapy.

### 2.2 Search strategy

PubMed, EMBASE.com, the Cochrane Central Register of Controlled Trials (CENTRAL), Chinese National Knowledge Infrastructure (CNKI), and Chinese Biological Medical Database (CBM), and ClinicalTrials.gov (http://clinicaltrials.gov/) will be searched. The search strategies have been developed by LG and reviewed by an experienced librarian researcher (KHY) to improve the search quality.^[25]^ The references of relevant systematic reviews/meta-analyses will be tracked to identify additional studies. The PubMed search strategies as follows,

- #1 “Stomach Neoplasms”[Mesh]
- #2 Digest^∗^[Title/Abstract] or Gastr^∗^[Title/Abstract] or gut[Title/Abstract] or epigastr^∗^[Title/Abstract] or stomach^∗^[Title/Abstract]
- #3 #1 or #2
- #4 “Adenocarcinoma”[Mesh]
- #5 carcin^∗^[Title/Abstract] or cancer^∗^[Title/Abstract] or neoplas^∗^[Title/Abstract] or tumour^∗^[Title/Abstract] or tumor^∗^[Title/Abstract] or cyst^∗^[Title/Abstract] or adenocarcin^∗^[Title/Abstract] or malig^∗^[Title/Abstract]
- #6 #4 or #5
- #7 “Gastrectomy”[Mesh]
- #8 gastrectomy[Title/Abstract]
- #9 operab[Title/Abstract]
- #10 gastrect^∗^[Title/Abstract]
- #11 resect^∗^[Title/Abstract]
- #12 #7 or #8 or #9 or #10 or #11
- #13 #3 and #6
- #14 #13 and #12

### 2.3 Study selection

Initial search records will be imported into the web-based systematic review software package “Rayyan”.^[26]^ First, the titles and abstracts of records will be reviewed independently to identify potential trials according to eligibility criteria. Then, full-text of all potentially relevant trials will be downloaded to make sure eligible trials. Any conflict will be resolved by discussion.

### 2.4 Data extraction

A standard data extraction form will be created using Microsoft Excel 2013 (Microsoft Corp, Redmond, WA, www.microsoft.com) to collect data of interest, which including general characteristics of included trials (e.g., name of first author, year of publication, whether single-center or multicenter, country of study, recruitment time frame, follow-up length, total sample size, inclusion, and exclusion criteria), details of participants (e.g., gender, age, resection margin status, percentage of total gastrectomy, percentage of R0, percentage of D2, AJCC/UICC stage), details of interventions (e.g., drug, dosage, route, and period of adjuvant therapy), and outcomes (OS, DFS, 5-years OS, 5-years DFS).

For survival outcomes, we will extract median OS and DFS, the reported HRs with corresponding 95% CIs. When HRs and/or 95% CIs are missing we will calculate them from Kaplan–Meier survival curves with the method described by Tierney and colleagues.^[27]^

Data extraction will be completed independently by paired reviewers. Any conflicts will be resolved by discussion.

### 2.5 Risk of bias of individual study

Paired reviewers will evaluate independently the risk of bias of included RCTs using a modified version of Cochrane tool,^[28,29]^ in which we will use response options of “Definitely or Probably Yes” (assigned a low risk of bias) and “Definitely or Probably No” (assigned a high risk of bias). The modified tool includes the method of random sequence generation, concealment of treatment allocation; blinding (participants, healthcare providers, data collectors, outcome assessors, and data analysts); infrequent missing outcome data and free from selective outcome reporting. We will use a threshold of >20% missing data as indicative of high risk of bias for missing data.

### 2.6 Geometry of the network

A network plot will be drawn to describe and present the geometry of the treatment network of comparisons across trials to ensure if a network meta-analysis is feasible. Trials will be excluded from network meta-analysis and just describe the findings of studies if the trials are not connected by treatments. Network geometry will use nodes to represent different chemotherapy regimens and edges to represent the head-to-head comparisons between network nodes. The size of nodes and thickness of edges are associated with sample sizes of intervention and numbers of included trials, respectively.^[30]^

We will categorize the network nodes as follows: surgery alone, S1, XELOX, S1 + paclitaxel, S1 + platinum, 5-Fu alone, etoposide + doxorubicin + cisplatin (EAP), epirubicin + formyltetrahydrofolate + 5-fluorouracil-based (ELF-based), formyltetrahydrofolate + 5-fluorouracil (LF), 5-fluorouracil + doxorubicin + mitomycin C (FMA), irinotecan-based, other 5-Fu-based two drugs, other 5-Fu-based 3 drugs.

### 2.7 Data synthesis

A Bayesian network meta-analysis will be performed using package “*gemtc*” package version 0.8–2 of R-3.4.1 software.^[31]^ The function *mtc.run* will be used to generate samples from using the Markov Chains Monte Carlo sampler. Four Markov Chains will be run simultaneously. We will set 10 000 simulations for each chain as the ‘burn-in’ period. Then posterior summaries are based on 100 000 subsequent simulations. The model convergence will be assessed using Brooks–Gelman–Rubin plots method.^[32]^

Pooled risk ratios (RRs) with 95% credible intervals (CrIs) will be calculated for 5-years OS and 5-years DFS. Pooled HRs with 95% CrI for OS and DFS. In addition, rank probabilities will be calculated, which indicates the probability for each treatment to be best, second best, etc. Finally, we will present pooled absolute effect differences for 5-years OS and 5-years DFS. To estimate absolute effect for 5-years OS and 5-years DFS, we will use median baseline risk from surgery alone and will apply it to the relative effect from the network meta-analysis.

Conventional meta-analyses will be conducted using a random effects model. Heterogeneity across head-to-head trials will be assessed using I^2^ statistics, which represents the proportion of heterogeneity that is not due to chance (but rather due to real differences across studies’ populations and interventions). The values of 25%, 50%, and 75% for the I^2^ as indicative of low, moderate, and high statistical heterogeneity, respectively. In addition, we will assess the global heterogeneity from the network meta-analysis models using the *mtc.anohe* command of the “*gemtc*” package.

Random effects and fixed effect models network meta-analyses will be conducted and the deviance information criterion (DIC) will be used to compare model fit and parsimony. The model with the lowest DIC will be preferred (differences >3 are considered meaningful). We will assess statistical inconsistency between direct and indirect evidence at the paired comparison level using node splitting method.^[33]^

We will also conduct subgroup analyses and network meta-regression analyses to explore statistical heterogeneity across trials and inconsistency between direct and indirect evidence. We will focus on following possible effect modifiers: gender, age, and percentage of total gastrectomy.

We will use STATA version 15.1 software (Stata Corporation, CollegeStation, TX) to draw a comparison-adjusted funnel plot to identify whether there will be a small sample effect among the networks.

### 2.8 Certainty of evidence

Two trained GRADE methodologists will use the GRADE framework to rate the certainty of evidence and classify evidence as high, moderate, low, or very low certainty. The starting point for certainty in estimates for RCTs is high but may be rated down based on limitations in risk of bias, imprecision, inconsistency, indirectness, and publication bias.^[34,35]^

We will rate the certainty of evidence for each direct comparison according to traditional GRADE guidance without imprecision domain for pairwise meta-analysis.^[36]^ The evidence for indirect will focus on the dominant first order loop, rating certainty of indirect evidence as the lowest certainty of the direct comparisons informing that dominant loop. In the absence of a first order loop, we will use a higher order loop to rate certainty in evidence and, similarly, we will use the lowest of the ratings of certainty for the direct estimates contributing to the loop. We will consider further rating down each indirect comparison for intransitivity if the distribution of potential effect modifiers is different in the 2 contributing direct comparisons. For the network estimate, we start with the certainty of evidence from the direct or indirect evidence that dominates the comparison and subsequently consider rating down our certainty in the network estimate for incoherence between the indirect and direct estimates. We also rate down the certainty of evidence if there is imprecision (wide CrIs) around the network estimates.^[37]^

## Author contributions

LG and KHY planned and designed the research; LG, YTW, LYH, and QXY tested the feasibility of the study; LG and JL wrote the manuscript; all authors approved the final version of the manuscript.

**Conceptualization:** Long Ge, Kehu Yang.

**Investigation:** Long Ge, Liangying Hou, Qingxia Yang, Yiting Wu, Jiang Li.

**Methodology:** Long Ge.

**Project administration:** Long Ge, Liangying Hou, Qingxia Yang, Yiting Wu, Xiue Shi, Jiang Li.

**Supervision:** Xiue Shi, Kehu Yang.

**Writing – original draft:** Long Ge, Jiang Li.

**Writing – review & editing:** Long Ge, Liangying Hou, Qingxia Yang, Yiting Wu, Xiue Shi, Jiang Li, Kehu Yang.
